# Resveratrol against 6-OHDA-induced damage of PC12 cells via PI3K/Akt

**DOI:** 10.1515/tnsci-2020-0165

**Published:** 2021-04-19

**Authors:** Nanqu Huang, Juan Huang, Ying Zhang, Mingji Chen, Jingshan Shi, Feng Jin

**Affiliations:** Drug Clinical Trial Institution, The Third Affiliated Hospital of Zunyi Medical University (The First People’s Hospital of Zunyi), No. 98, Fenghuang Road, Zunyi 563000, Guizhou, China; Department of Pharmacology and Chemical Biology, Institute of Medical Sciences, Shanghai Jiao Tong University School of Medicine, No. 280, South Chongqing Road, Shanghai 200025, China; Key Laboratory of Basic Pharmacology and Joint International Research Laboratory of Ethnomedicine of Ministry of Education, Zunyi Medical University, No. 6, Xuefu West Road, Xinpu New District, Zunyi 563000, Guizhou, China

**Keywords:** 6-hydroxydopamine, resveratrol, phosphoinositide 3-kinase, Parkinson’s disease, protein kinase B

## Abstract

**Objective:**

Our previous *in vivo* study found that resveratrol (Res), which is a phytoalexin, attenuated 6-hydroxydopamine (6-OHDA)-induced motor dysfunction by activating the phosphatidylinositol 3-kinase/protein kinase B (PI3K/Akt) signaling pathway in rats. Therefore, we further explored the protective effect of Res on 6-OHDA-induced damage to PC12 cells *in vitro* with respect to the PI3K/Akt signaling pathway.

**Methods:**

We incubated PC12 cells with 75 μM 6-OHDA for 24 h, and Res was then added at a final concentration of 25 μM; the protective effect was examined via MTT and lactate dehydrogenase (LDH) assays. In addition, the PI3K inhibitor LY294002 was used to investigate the potential mechanism. JC-1 staining was used to detect the mitochondrial membrane potential (MMP), and western blotting (WB) was used to detect the phosphorylation of Akt-Ser473.

**Results:**

Compared with that in the control, the cell viability, total superoxide dismutase (SOD) activity, MMP, and p-Akt-Ser473 level of 6-OHDA-treated PC12 cells were significantly decreased, while the leakage rate of LDH was increased. And after treatment with 25 μM Res, the cell viability, total SOD activity, MMP, and p-Akt-Ser473 level of 6-OHDA-treated PC12 cells were significantly increased compared with those of the control cells, while the leakage rate of LDH was decreased. These effects of Res were antagonized by LY294002.

**Conclusions:**

Res ameliorates 6-OHDA-induced damage to PC12 cells via activation of the PI3K/Akt signaling pathway.

## 1 Introduction

Parkinson’s disease (PD) is a neurodegenerative disease common in middle-aged and elderly people characterized by resting tremors, muscle stiffness, decreased movement, and abnormal posture and gait. Although PD is usually not directly fatal, the motor dysfunction caused by PD seriously affects the self-care ability of middle-aged and elderly people and seriously reduces the quality of life of patients [1,2]. Therefore, actively exploring the pathogenesis and therapeutic drugs of PD has important scientific significance and social value. With the deepening of people’s understanding of PD, an increasing number of studies have shown that abnormal signaling pathways caused by oxidative stress injury play a vital role in the occurrence and development of PD [3,4]. Pathological examination revealed that Akt phosphorylation in the substantia nigra pars compacta (SNc) of PD patients was significantly reduced, and there were a large number of apoptotic dopaminergic (DA) neurons [5]. *In vitro*, oxidative stress-induced mitochondrial dysfunction can be inhibited by the antioxidant allicin by upregulating phosphatidylinositol 3-kinase/protein kinase B (PI3K/Akt) [6]. *In vivo*, the antioxidant Danshensu can improve rotenone-induced motor dysfunction in mice by upregulating the PI3K/Akt pathway [7]. Our previous *in vivo* study found that resveratrol (Res), which is a phytoalexin, improved 6-hydroxydopamine (6-OHDA)-induced motor dysfunction by activating the PI3K/Akt signaling pathway in rats [8]. Therefore, we further explored the protective effect of Res on 6-OHDA-induced damage to PC12 cells *in vitro*.

## 2 Material and methods

### 2.1 Culture and treatment of cells

PC12 cells were obtained from the American Type Culture Collection (Rockville, MD, USA) and were cultured routinely in RPMI 1640 medium (SH30809.01, Thermo Fisher Scientific) supplemented with 10% horse serum (E500007, Sangon Biotech), 5% fetal bovine serum (16000-044, Gibco), 100 U/mL benzyl penicillin, and 100 mg/L streptomycin (P1400, Solarbio) at 37°C in a humidified 5% CO_2_ atmosphere. The cells were seeded into 96-well plates, 24-well plates, or 6-well plates at a density of 1.5 × 10^5^ cells/mL for 24 h. Stock solutions of 6-OHDA (H4381, Sigma-Aldrich) were freshly prepared with L-ascorbic acid (0.2%, A5960, Sigma-Aldrich). Unless otherwise stated, PC12 cells were incubated with Res (≥98%, Zelang Medical Technology) for 2 h followed by treatment with 75 μM 6-OHDA for an additional 24 h before further experiments, and the PI3K inhibitor LY294002 (S1737, Beyotime Biotechnology) was added 0.5 h before Res. The experiments were repeated at least three times in different batches of cells.


**Ethical approval:** The conducted research is not related to either human or animals use.

### 2.2 Cell viability assay

Cell viability was determined using a thiazolyl blue tetrazolium bromide (MTT, ST316, Beyotime Biotechnology) assay. Briefly, PC12 cells were cultured in 96-well plates. After incubation, 5 mg/mL MTT solution (prepared in 1× PBS) was added to each well, and then the cells were incubated at 37°C in a 95% air/5% CO_2_ atmosphere for 4 h. After removing the medium with MTT, 100 µL DMSO (D8371, Solarbio Life Science) was added to each well and shaken at room temperature for 10 min. Then, the optical density at 570 nm (OD570) was measured by a microplate reader (Multiskan GO, Thermo Scientific, USA).

### 2.3 Measurement of mitochondrial membrane potential (MMP)

As a major determinant of early apoptosis, changes in the MMP were measured by using a JC-1 kit (C2006, Beyotime Biotechnology) according to the manufacturer’s instructions. After PC12 cells were treated with different compounds, the medium was removed, and the cells were incubated with 10 µM JC-1 (prepared in RPMI 1640) at 37°C for 30 min. Carbonyl cyanide *m*-chlorophenyl hydrazone (CCCP), a mitochondrial electron transport chain inhibitor, was added to the MMP-decreased control group 30 min before JC-1 addition. After this, the cells were washed with PBS and observed under an inverted fluorescence microscope (IX73, Olympus, Japan).

### 2.4 Lactate dehydrogenase (LDH) and total superoxide dismutase (SOD)

LDH (C0016, Beyotime Biotechnology) release is considered to be an important indicator of cell membrane integrity and is widely used in cytotoxicity assays. The changes in the LDH leakage rate were measured using an LDH kit according to the manufacturer’s protocol. PC12 cells were seeded into a 96-well plate, and the absorbance of the samples was measured at 490 nm using a microplate reader (Multiskan GO, Thermo Scientific, USA). The SOD activity of PC12 cells was measured using a total SOD assay kit (S0101, Beyotime Biotechnology) with WST-8 according to the manufacturer’s protocol. PC12 cells were seeded into a 6-well plate, and the absorbance of the samples was measured at 450 nm using a microplate reader (Multiskan GO, Thermo Scientific, USA).

### 2.5 Western blot analysis

Briefly, the treated PC12 cells were lysed and homogenized in RIPA lysis buffer (P0013B, Beyotime Biotechnology) supplemented with proteinase inhibitor phenylmethylsulfonyl fluoride (PMSF) (ST505, Beyotime Biotechnology) and 1× phosphatase inhibitor cocktail (P1260, Solarbio). The lysates were treated on ice for 30 min and centrifuged at 37°C and 14,000 g for 15 min. Then, the whole protein levels of the supernatant were quantified by a BCA assay kit (GK5012, Generay). Equal amounts of total protein (10 µg per lane). Then, the lysates were separated using 8–12% SDS-PAGE (P0012AC, Beyotime Biotechnology), and the proteins were transferred to PVDF (IPVH00010, Merck) membranes. After membranes were blocked with 5% nonfat milk in TBST (0.1% Tween 20) at room temperature for 1 h, membranes were incubated with Akt (9272S, CST, 1:1,000) and p-Akt-Ser473 (4060P, CST, 1:1,000) and then incubated with goat anti-rabbit and mouse IgG-HRP (M21003, Abmart, 1:2,000). After that, ECL (E002-100, 7seabiotech) reagent was used to visualize the protein-antibody complexes. Quantity Ones software (Bio-Rad) was used to quantitate the intensity of the band.

### 2.6 Statistical analysis

All statistical analyses were performed with SPSS 22.0 (IBM, USA). All results are expressed as the mean ± standard error of the mean (SEM). The means of more than two groups were analyzed by one-way analysis of variance (ANOVA). When ANOVA showed significant differences, pairwise comparisons between means were assessed by Bonferroni’s *post-hoc t*-test with correction. A value of *P* < 0.05 was considered statistically significant.

## 3 Results

### 3.1 Protective effect of Res on 6-OHDA-induced PC12 cell injury

**Figure 1:**
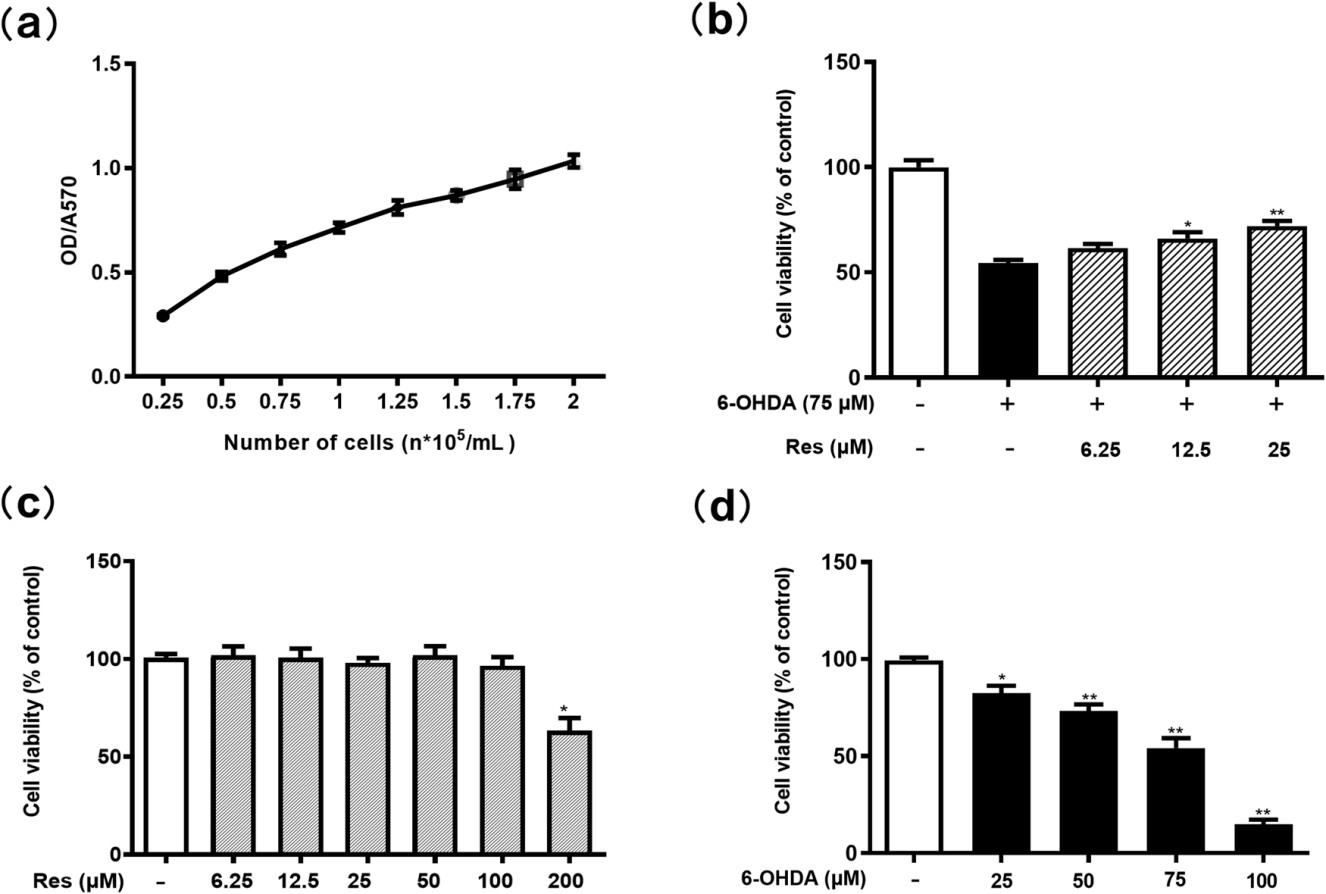
Protective effect of Res on 6-OHDA-treated PC12 cells. (a) PC12 cell growth curve. (b) Effects of Res simple pretreatment on PC12 cells (*P < 0.05 vs control). (c) Effects of different concentrations of 6-OHDA on PC12 cell activity (*P < 0.05, **P < 0.01 vs control). (d) Effects of Res on 6-OHDA-induced growth inhibition in PC12 cells (*P < 0.05, **P < 0.01 vs 6-OHDA group) (mean ± SEM, *n* = 4).

PC12 cell injury was measured by the MTT method after 48 h of culture. The OD570 for 1.5 × 10^5^/mL cells was 0.86 ± 0.05 in a good linear range, indicating an appropriate cell seeding density (Figure 1a). Incubation with different concentrations of Res for 24 h revealed no significant toxicity up to 200 μM. With reference to our previous work, we chose 25 μM Res as the maximum dose [9] (Figure 1b). After PC12 cells were incubated with different concentrations of 6-OHDA for 24 h, the cell viability decreased with increasing 6-OHDA concentration. The cell viability decreased to 54.20 ± 4.38% (*P* < 0.05 vs Control) at a final concentration of 75 μM, which was chosen as a model concentration (Figure 1c). It was found that 12.5 μM and 25 μM Res significantly improved cell viability (*P* < 0.05 vs Model) in a dose-dependent manner (Figure 1d).

### 3.2 PI3K inhibitor weakens the protective effect of Res on 6-OHDA-induced PC12 cell damage

**Figure 2:**
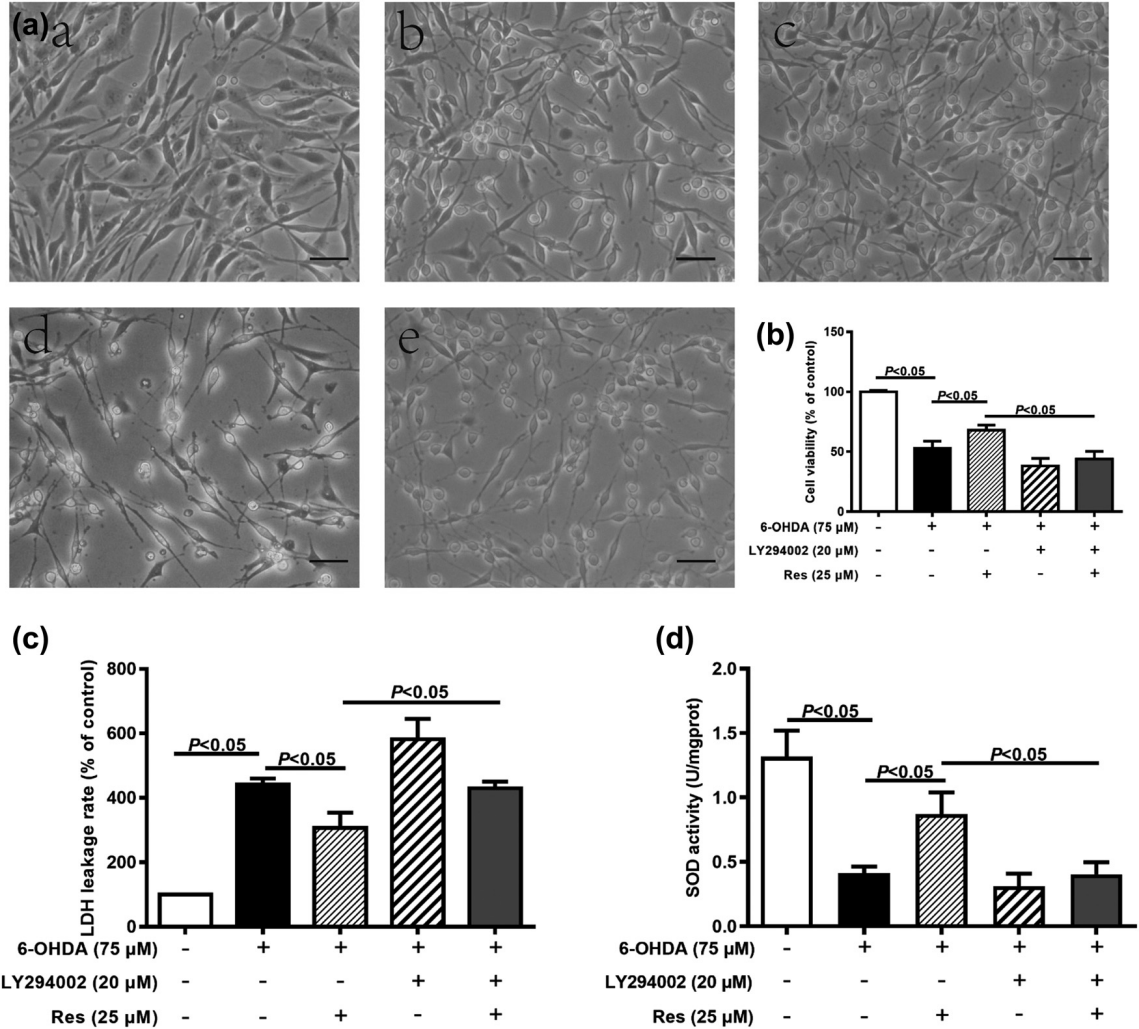
Representative pictures of PC12 cells (scale bar is 20 μm). (a(a)) Control group, (a(b)) 6-OHDA group, (a(c)) 6-OHDA + Res-25 μM group, (a(d)) 6-OHDA + LY group, (a(e)) 6-OHDA + Res-25 μM + LY group. (b) PC12 cell viability. (c) LDH leakage rate. (d) Total SOD activity (mean ± SEM, *n* = 4).

Compared with that in the control group, the number of cells in the 6-OHDA group was reduced (Figure 2a(b)). The 6-OHDA + Res-25 μM group had more cells than 6-OHDA group (Figure 2a(c)). The number of cells in the 6-OHDA + Res-25 μM + LY294002 group was similar to that in the 6-OHDA + LY294002 group (Figure 2a(e)), showing that LY294002 weakens the protective effect of Res on 6-OHDA-induced PC12 cell damage. The viability of PC12 cells determined by MTT showed the same result (Figure 2b).

### 3.3 LDH and total SOD

LDH is a soluble cytoplasmic enzyme that can represent the degree of cell damage to a certain extent. As shown in Figure 2c, Res significantly reduced the leakage rate of LDH in PC12 cells. SOD is an important member of the antioxidant enzyme system in biological systems, as shown in Figure 2d. Res significantly increased total SOD activity in PC12 cells. Interestingly, these effects of Res were antagonized by LY294002, as shown in Figure 2c and d.

### 3.4 MMP

**Figure 3:**
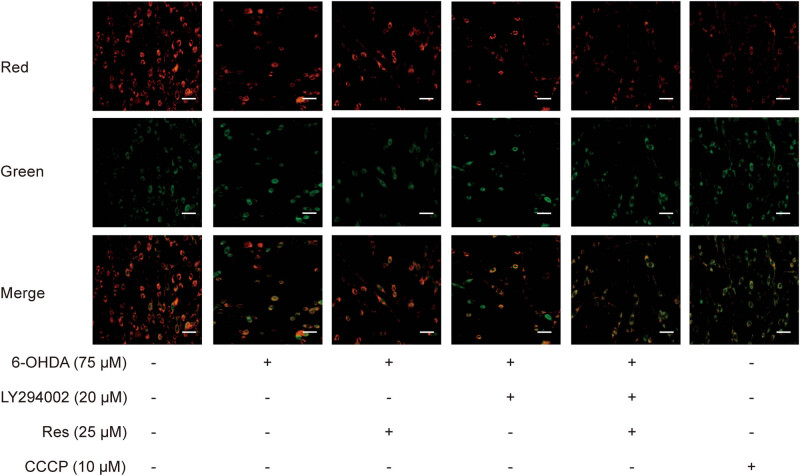
Res increased the MMP of 6-OHDA-induced PC12 cells. Red and green fluorescence represent higher and lower MMP, and CCCP was used as a control for the depolarization of the mitochondria (scale bar: 20 μm, *n* = 4).

**Figure 4:**
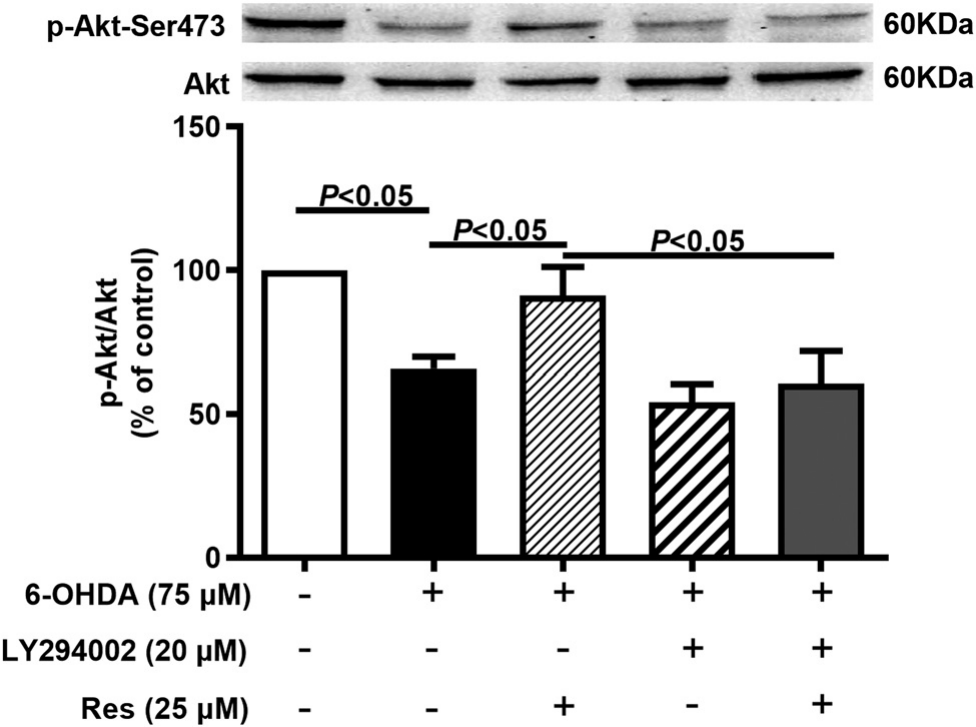
Res increased the expression of p-Akt-Ser473 in 6-OHDA-treated PC12 cells. The p-Akt-Ser473/Akt (mean ± SEM, *n* = 4).

Normally, cells maintain stable levels of intracellular MMP, and this stability is thought to be a requisite for normal cell functioning. In this study, we use JC-1 to detect the MMP. In normal cells, JC-1 aggregates in mitochondria and emits red fluorescence, while in damaged cells, JC-1 exists as a green fluorescence monomer that accrues in the cytosol. As shown in Figure 4, the control group had strong red fluorescence and weak green fluorescence. After incubation with 6-OHDA for 24 h, the red fluorescence was enhanced and the green fluorescence became faint, which was consistent with the CCCP group. Compared with that of the 6-OHDA group, the red fluorescence of the 25 μM Res group was enhanced and the green fluorescence was decreased, indicating that the MMP was increased. At the same time, the fluorescence of the LY group was similar to that of the CCCP group. Inhibition of the PI3K/Akt signaling pathway decreased the effect of Res on the MMP (Figure 3).

### 3.5 Res increased the expression of p-Akt-Ser473 in 6-OHDA-treated PC12 cells

Ser473 is the major phosphorylation site of Akt, so we detected it in 6-OHDA-treated PC12 cells. As shown in Figure 4, the level in the 6-OHDA group was significantly lower than that in the control group, while Res increased the expression of p-Akt-Ser473. Interestingly, these effects of Res were antagonized by LY294002.

## 4 Discussion

In the present study, we found that Res has a protective effect in 6-OHDA-induced PC12 cell damage. It can increase total SOD activity, increase MMP, and increase the level of Akt-Ser473 phosphorylation. Blocking PI3K with inhibitors can antagonize this protective effect.

The pathogenesis of PD is complex, and oxidative stress is an important pathogenic factor [3,4]. Therefore, this study used PC12 cells, which have many similar characteristics to DA neurons, and applied dopamine analogs and the neurotoxin 6-OHDA, which can produce oxidative stress *in vitro* and *in vivo,* to simulate DA neuron damage caused by oxidative stress *in vitro*. It is well-known that oxidative stress is an imbalance caused by excessive ROS produced by mitochondrial oxidative phosphorylation and/or a weakened antioxidant system [10]. It can be simply understood as excessive ROS production or reduced clearance. The main source of ROS is mitochondria, and damage to mitochondria can lead to excessive ROS production [11]. However, the main target of ROS attack is also mitochondria. Therefore, these excessively generated ROS induce oxidative damage to mitochondrial DNA, and this oxidative damage further aggravates mitochondrial damage and eventually leads to the death of DA neurons [12]. In the process of respiration and oxidation, mitochondria store the energy generated in the inner mitochondrial membrane as electrochemical potential energy, which causes the asymmetric distribution of the concentration of protons and other ions on both sides of the inner membrane to generate the MMP [13]. Therefore, the MMP reflects the integrity of mitochondrial function, so in this study, we reflected the integrity of mitochondrial function by detecting the MMP. We found that Res increased the MMP of PC12 cells damaged by 6-OHDA (Figure 3). This indicates that Res has a certain protective effect on mitochondrial damage caused by excessive ROS production. The other main factor for excessive ROS is the reduction in ROS clearance, which is mainly manifested as the weakening of the antioxidant system with SOD as the main component [14]. SOD is an important antioxidant enzyme. Due to its special molecular structure, SOD can give an electron to ROS, turning ROS into harmless substances, but SOD itself will not form harmful substances [15]. Therefore, this study assessed the effect of Res on the antioxidant system of PC12 cells damaged by 6-OHDA by detecting the total SOD activity. We found that Res significantly increased the total SOD activity of cells (Figure 2d), indicating that Res can restore the ability of cells to resist oxidation and eliminate ROS, thereby playing a protective role. Overall, in this experiment, Res exerted a neuroprotective effect against oxidative stress by inhibiting the generation and elimination of ROS.

PI3K/Akt is a very important signaling pathway in neuronal development, survival, and energy metabolism, and it is closely related to protection against neuronal oxidative stress [16]. Its antioxidative stress effect is mainly manifested in the following two aspects. On the one hand, it protects the function of mitochondria, inhibits the mitochondrial apoptosis pathway, and prevents the death of an abnormally large number of neurons caused by the vicious cycle of oxidative stress [17]. On the other hand, it activates downstream signaling pathways such as nuclear factor E2-related factor 2 (Nrf2) to produce a large amount of antioxidant products, such as SOD and heme oxygenase 1 (HO-1), thereby exerting an antioxidative stress effect [18]. Therefore, the PI3K/Akt signaling pathway is the key to neuron resistance to oxidative stress, and activation of this pathway can play a neuroprotective effect [17,18]. The research group found that Res can attenuate the motor dysfunction caused by oxidative stress in rats. The preliminary mechanism is related to the inhibition of DA neuron apoptosis and the activation of the PI3K/Akt signaling pathway [8]. However, in previous *in vivo* experiments, we did not inhibit or block the PI3K/Akt signaling pathway, nor did we detect some indicators related to the generation and removal of ROS by Res. It is only a preliminary study.

Therefore, in this *in vitro* study, we used a PI3K inhibitor (LY294002) to inhibit PI3K to clarify the mechanism. We found that inhibiting PI3K weakened the protective effect of Res on 6-OHDA-induced cell damage by affecting factors including cell viability, MMP, and total SOD activity (Figures 2–4). This indicates that the PI3K/Akt signaling pathway is the key pathway by which Res attenuates the damage caused by oxidative stress. Res can promote the elimination of ROS by activating the PI3K/Akt signaling pathway and can inhibit the generation of ROS, thereby reducing the damage to DA neurons caused by oxidative stress. However, this study also has certain limitations. For example, we did not further test mitochondrial function, nor did we directly detect the generation of ROS; instead, we assessed MMP to highlight this effect.

## 5 Conclusion

These results suggest that Res ameliorates 6-OHDA-induced damage to PC12 cells via activation of the PI3K/Akt signaling pathway.
